# A Lagerstätte from Australia provides insight into the nature of Miocene mesic ecosystems

**DOI:** 10.1126/sciadv.abm1406

**Published:** 2022-01-07

**Authors:** Matthew R. McCurry, David J. Cantrill, Patrick M. Smith, Robert Beattie, Mary Dettmann, Viktor Baranov, Charles Magee, Jacqueline M. T. Nguyen, Marnie A. Forster, Jack Hinde, Ross Pogson, Helen Wang, Christopher E. Marjo, Paulo Vasconcelos, Michael Frese

**Affiliations:** 1 Australian Museum Research Institute, 1 William Street, Sydney, New South Wales 2010, Australia.; 2 Earth and Sustainability Science Research Centre, School of Biological, Earth and Environmental Sciences (BEES), University of New South Wales, Kensington, New South Wales 2052, Australia.; 3 Paleobiology, National Museum of Natural History, Smithsonian Institution, Washington, DC 20560, USA.; 4 Royal Botanic Gardens Victoria, Private Bag 2000, South Yarra, Victoria 3141, Australia.; 5 Department of Biological Sciences, Macquarie University, North Ryde, New South Wales 2109, Australia.; 6 Geosciences, Queensland Museum, South Brisbane, Queensland 4101, Australia.; 7 Ludwig-Maximilian University of Munich, Biocenter, Großhaderner Strasse 2, 82152 Planegg-Martinsried, Germany.; 8 Geoscience Australia, Symonston 2609, Australian Capital Territory, Australia.; 9 College of Science and Engineering, Flinders University, Bedford Park, South Australia 5042, Australia.; 10 Research School of Earth Sciences, Australian National University, Canberra, Australian Capital Territory 0200, Australia.; 11 Illawarra Environmental Education Centre, Shell Cove, New South Wales 2529, Australia.; 12 Mark Wainwright Analytical Centre, University of New South Wales, Kensington, New South Wales 2052, Australia.; 13 School of Earth Sciences, The University of Queensland, Brisbane, Queensland 4072, Australia.; 14 Commonwealth Scientific and Industrial Research Organisation, Health and Biosecurity, Black Mountain, Australian Capital Territory 2601, Australia.; 15 Faculty of Science and Technology, University of Canberra, Bruce, Australian Capital Territory 2601, Australia.

## Abstract

Reduced precipitation in the Miocene triggered the geographic contraction of rainforest ecosystems around the world. In Australia, this change was particularly pronounced; mesic rainforest ecosystems that once dominated the landscape transformed into the shrublands, grasslands, and deserts of today. A lack of well-preserved fossils has made it difficult to understand the nature of Australian ecosystems before the aridification. Here, we report on an exceptionally well-preserved rainforest biota from New South Wales, Australia. This Konservat-Lagerstätte hosts a rich diversity of microfossils, plants, insects, spiders, and vertebrate remains preserved in goethite. We document evidence for several species interactions including predation, parasitism, and pollination. The fossils are indicative of an oxbow lake in a mesic rainforest and suggest that rainforest distributions have shifted since the Miocene. The variety of fossils preserved, together with high fidelity of preservation, allows for unprecedented insights into the mesic ecosystems that dominated Australia during the Miocene.

## Teaser

The discovery of an exceptionally well-preserved fossil site provides new insight into the nature of Miocene ecosystems.

## INTRODUCTION

The Miocene epoch [23.03 to 5.33 million years (Ma) ago] saw a remarkable change in global ecosystems. Temperatures increased in the early to middle Miocene, before undergoing a marked drop, which triggered a global extinction event known as the Middle Miocene disruption (*1*). In Australia, the environment became progressively drier, leading to an extensive contraction of the rainforests that once covered much of the continent (*2*–*4*). This process shaped many of the biotic communities found in modern-day Australia (*3*). Rainforest plants gave way to more dryland-adapted species, e.g., *Casuarina* and *Eucalyptus*, and the diversity of shrubs and grasses that now characterize the Australian flora.

Fossil localities such as Alcoota and Bullock Creek in the Northern Territory and Riversleigh in Queensland preserve an exceptional diversity of large vertebrates from the Miocene in extensive bone beds (*5*–*7*). However, the fossil record of insects, spiders, and other soft-bodied organisms from the Miocene of Australia is comparatively poor (*8*). The scarcity of Cenozoic Lagerstätten has made it difficult to interpret how changes in the climate contributed to the evolution of Australia’s biota and the development of modern ecosystems.

Here, we report on the discovery of a new fossil Lagerstätte in central New South Wales, named McGraths Flat (sans apostrophe, following guidelines for Australian place names) after N. McGrath who found the fossil site. The site preserves soft tissues from a wide variety of plants and animals and provides a unique opportunity to study the flora and fauna of Australia during the Miocene aridification of the continent. This report is the first account of the site and includes details of the geological context, paleoenvironmental setting, examples of preserved plants and animals, and an evaluation of the fidelity of preservation.

## RESULTS

### Geology and age

**Fig. 1.**
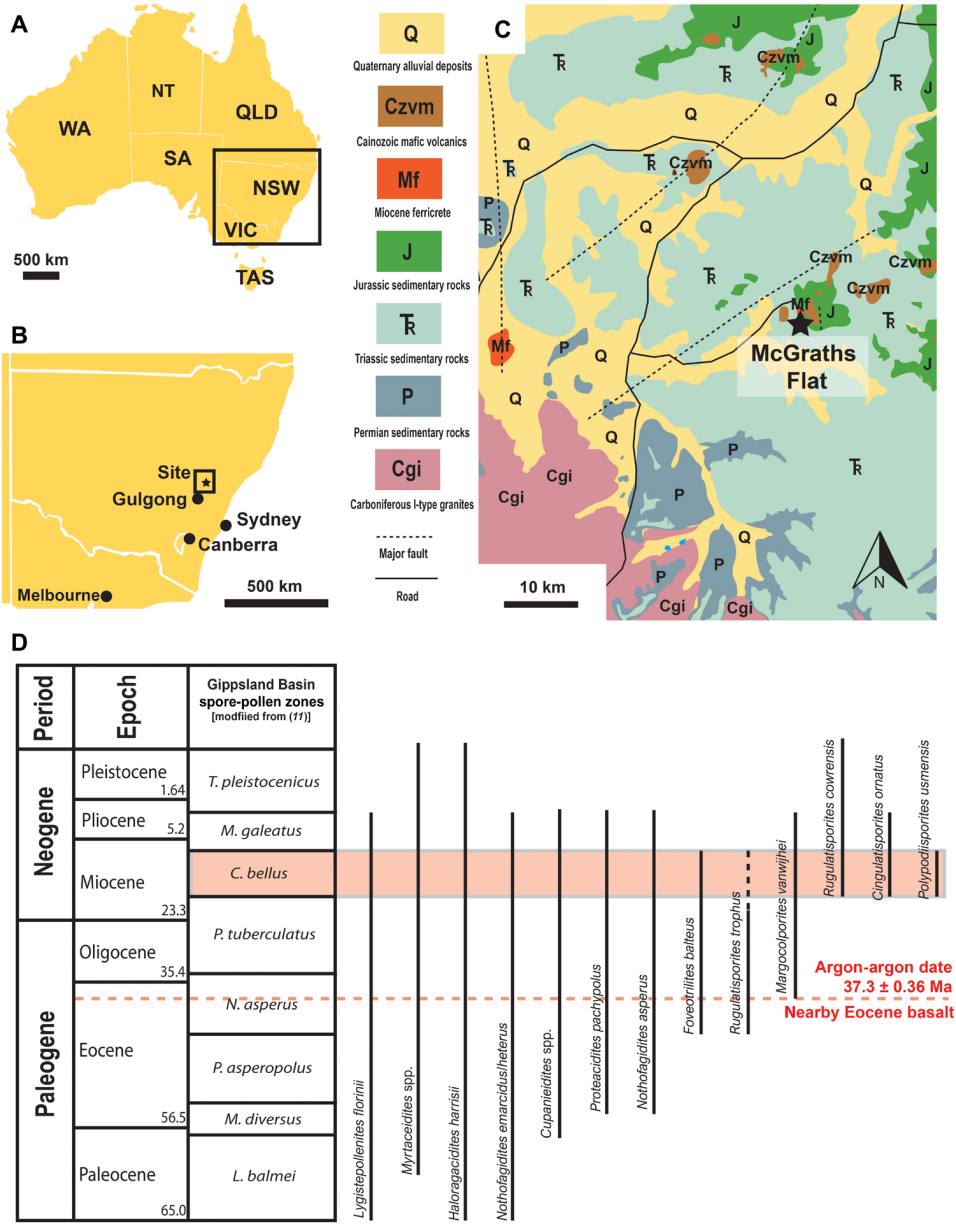
Location and geology. (**A** and **B**) Location of the site in New South Wales, Australia. (**C**) Local geology near the site showing Miocene ferricrete and extensive Mesozoic bedrock. (**D**) Temporal ranges of biostratigraphically informative spores and pollen found at the site suggest a mid-Miocene age (red box that includes the *C. bellus* Zone). The red dotted line indicates the ^40^Ar/^39^Ar age of the nearby Eocene basalt. WA, Western Australia; NT, Northern Territory; SA, South Australia; QLD, Queensland; NSW, New South Wales; VIC, Victoria; TAS, Tasmania.

McGraths Flat is situated on a private property in the Central Tablelands of New South Wales, ~25 km northeast of Gulgong (Fig. 1, A to C). The fossiliferous stratum is exposed as loose blocks moved to the surface via agricultural tilling, and, even at depth, the bed is highly fractured. Excavation revealed that the blocks likely originated from a single 300- to 500-mm-thick layer consisting of finely bedded goethite FeO(OH), with both fossils and matrix composed of the same material (Supplementary Methods, figs. S1 and S2, and table S1). The goethite bed is directly underlain by an ~8-cm-thick sandstone layer that overlays a conglomerate. The fossiliferous layer is restricted to a small geographic area (1000 to 2000 m^2^). Flanking the deposit are hills capped by basalt that outcrop 70 m to the east and 420 m to the west (fig. S3). We infer these basalts as the source of iron composing the goethite, a hypothesis that is consistent with the observation of other ferricrete deposits in the region that overlie nearby basalts (*9*). In such a scenario, dissolved iron would only need to have traveled 3 to 5 m vertically downward through the soil column in solution to reach the area of deposition. In a rainforest environment, the decay of organic matter often causes groundwaters to become acidic and reducing, which aids in the transport of dissolved iron (*10*).

The presence of palynofloras consistent with those of the *Canthiumidites bellus* Zone, as defined by Stover and Partridge (*11*) for the Gippsland Basin and modified by Macphail (*12*, *13*) for the Murray Basin, indicates a latest early to middle Miocene age (~11 to 16 Ma) for the fossiliferous deposit. Attribution of the sediments to this zone is based on the presence of *Cingulatisporites ornatus*, *Polypodiisporites usmensis*, *Foveotrilites balteus*, and *Rugulatisporites cowrensis* in association with frequently occurring *Nothofagidites*, *Haloragacites*, and myrtaceous pollen (Fig. 1D and fig. S2). The Australian palynozonation is well constrained, with many ties to radiometrically dated sites and sites aged through dinoflagellate and foraminiferal biostratigraphy; thus, palynozonation provides a reliable means to date this locality (*11*, *12*). ^40^Ar/^39^Ar geochronology of the nearby basalts and dating of detrital zircons within the deposit provided additional maximum constraints on the age of the fossils (Supplementary Methods, fig. S4, and table S2).

### Depositional setting and mode of preservation

The fine bedding of the goethite matrix, contrasted against the coarser sandstones and conglomerates underneath the deposit, indicates that the fossils may have been deposited in a low-energy water body, but that the site was a higher-energy water channel before this. A high abundance of immature phantom midges (*Chaoborus*, Chaoboridae, and Diptera), normally found only when predator populations are absent or low, suggests that the depositional environment was isolated enough that aquatic predators could not easily enter and leave the environment (*14*). However, the presence of the occasional fish and a variety of rheophilic insects indicates that the water body at McGraths Flat maintained some connection to a river channel, likely through overbank flows. The limited geographic distribution of the deposit, combined with the low number of larger vertebrate predators, strongly suggests that the environment of deposition was relatively small and local in scale, similar to that of an oxbow lake.

The bedding planes are present as layers wrapping around inclusions in the matrix. This implies that the matrix may have formed as a precipitate around the fossils before compaction (fig. S5). Dissolved iron (Fe^2+^) likely leached during weathering of basalt to saprolite, traveled downward to the water table, and entered the water body through lateral seepages. In the oxbow lake, the iron underwent oxidation to Fe^3+^ and precipitated as fine layers of goethite at the bottom of the water body, encasing plant leaves, insects, and other organisms. Iron precipitation may have been triggered by intermittent torrential rains that would bring oxygenated waters or by supersaturation due to evaporation when waters would become more alkaline. In the McGraths Flat deposit, the fossils have been both encased and replaced by goethite. This mode of preservation is analogous to the iron cementation of bacteria and fungi that has been observed in suspended lakes and in the groundwater system of weathering banded iron formations (*15*). It has been suggested that iron mineral incrustation of microorganisms begins while the organisms are still alive (*16*).

### Flora and fauna

**Fig. 2.**
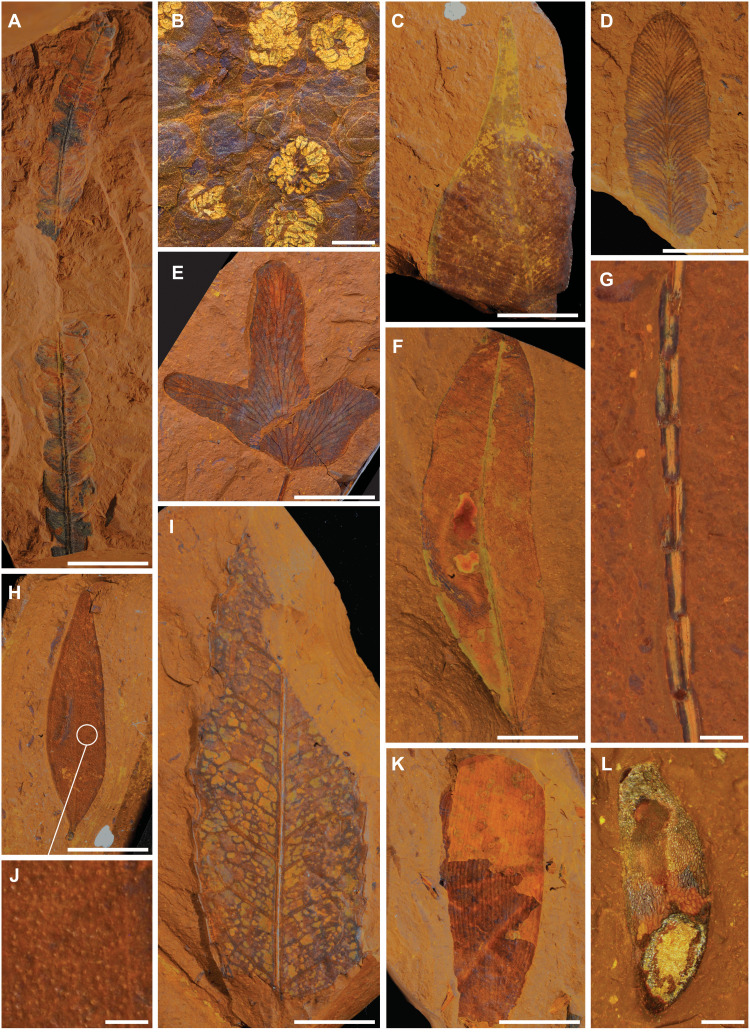
Plant macrofossils. (**A**) *Banksia* sp. (AM F.146586); (**B**) isolated flowers thought to be affiliated with Malvales (AM F.146587); (**C**) entire-margin leaf apex with prominent drip tip (AM F.146588); (**D** and **E**) fern pinnules assigned to *Lygodium* sp. (AM F.146589 and AM F.146600, respectively); (**F**) myrtaceous leaf with prominent intramarginal vein and numerous oil glands (AM F.146591); (**G**) isolated twig of *Gymnostoma* sp. (AM F.146590); (**H**) different type of myrtaceous leaf with prominent oil glands (AM F.146592); (**I**) large toothed leaf with semi-craspedodromous venation (AM F.146593); (**J**) close-up of the oil glands of AM F.146592; (**K**) *Agathis* sp. (AM F.146603); and (**L**) isolated winged samara of uncertain affinities (AM F.146604). Scale bars, 1 mm (B, G, J, and L) and 1 cm (A, C to F, H, I, and K).

McGraths Flat preserves an unusually high diversity of plants and animals. The flora of McGraths Flat is generally consistent with that of a mesic rainforest environment. However, the presence of some sclerophyllous elements and pollen that are normally associated with drier environments indicate that the rainforest was close to other habitats (Fig. 2, fig. S2, and table S3). Leaves range from notophyll to mesophyll in size, and many of the larger leaf forms exhibit morphology that indicates rainforest habitat. These morphologies include prominent drip tips (Fig. 2C), entire leaf margins, and large leaf sizes (up to 400 mm long). Sclerophyllous elements include *Banksia* and some myrtaceous material that were likely transported from rainforest fringes or drier sites away from the main water body (Fig. 2, A, F, H, and J).

To date, 50 angiosperm leaf taxa containing a mixture of entire marine and toothed margin forms have been recognized (Fig. 2I). Ferns are represented by pinnules, isolated sorophores, spores of *Lygodium* (Fig. 2, D and E), fertile segments of *Gleichenia*, and a possible fertile filmy fern. However, the number of fern spore microfossils (14 taxa) indicates a much greater diversity, including Blechnaceae, Cyatheaceae, Dicksoniaceae, and Thyrsopteridaceae. The spore flora also reflects the presence of lycopods and bryophytes, including Hepaticae. Conifers are represented by both leaves (*Agathis*) and pollen of Araucariaceae (*Araucariacites* and *Dilwynites*) (Fig. 2K). The presence of Podocarpaceae has been detected in the pollen record as *Podocarpidites* (*Podocarpus*) and *Lygistepollenites* (*Dacrydium*) and in a small number of podocarp leaves. The rarity of leaves may indicate that podocarpaceous plants were not a large component of the vegetation immediately adjacent to the depositional site. Most leaf fossils are angiosperms and include Casuarinaceae (*Gymnostoma*) (Fig. 2G), Proteaceae (*Banksia*), Myrtaceae (three leaf types), Lauraceae (*Cryptocarya*), Malvaceae (cf. *Argyrodendron* and *Brachychiton*), Cunoniaceae (cf. *Ceratopetalum*), and Nothofagaceae (*Nothofagus*) (fig. S6 and table S3). The most frequently found pollen is *Nothofagidites* representing *Nothofagus* (both *Lophozonia* and *Brassospora*). Other families that present as pollen but not identified in the leaf flora include probable caesalpinioid Fabaceae, Paracryphiaceae (*Quintinia*), Sapindaceae, Santalaceae, Restionaceae, and Sparganiaceae. Flowers, fruits, and seeds are also present in the deposit (Fig. 2B).

**Fig. 3.**
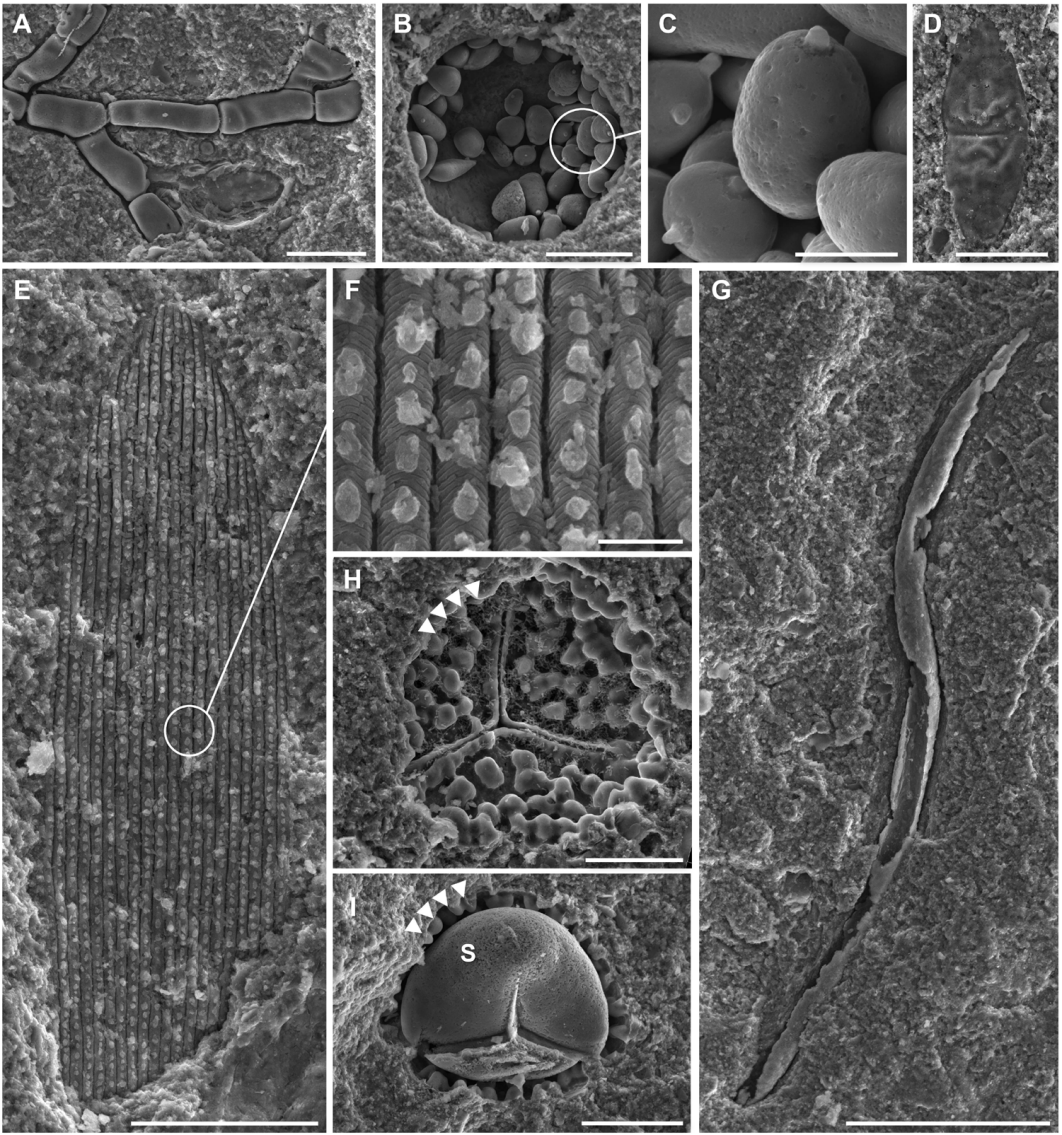
Miscellaneous microfossils. (**A**) Fungal hypha (AM F.146156.2); (**B** and **C**) yeast-like fungal cells (AM F.145847.1); (**D**) *Dicellasporites* sp. (AM F.145716.1); (**E** and **F**) lepidopteran wing scale (AM F.146089.1); (**G**) nematode (AM F.145716.2); (**H**) *Rugulatisporites trophus* (AM F.145717.1) showing the imprint of exine (arrowheads); and (**I**) *Rugulatisporites* sp. with both exine (arrowheads) and intine steinkern (S) (AM F.146155.1). Scale bars, 2 μm (F), 5 μm (D), 10 μm (H and I), 20 μm (A, B, and E), and 50 μm (C and G).

A variety of microfossils have also been found at McGraths Flat. Fungi are common in the deposit as fungal spores (*Dactylaria*, *Dicellaesporites*, and *Dyadosporites*), hyphae and occasional asci of Ascomycetes, and yeast (Basidiomycota) (Fig. 3, A to D). The fungi are often preserved with detailed features, e.g., yeast cells frequently show birth and bud scars (Fig. 3, C and D). Nematodes and a glochidium (parasitic bivalve larva) have also been found (Fig. 3G).

**Fig. 4.**
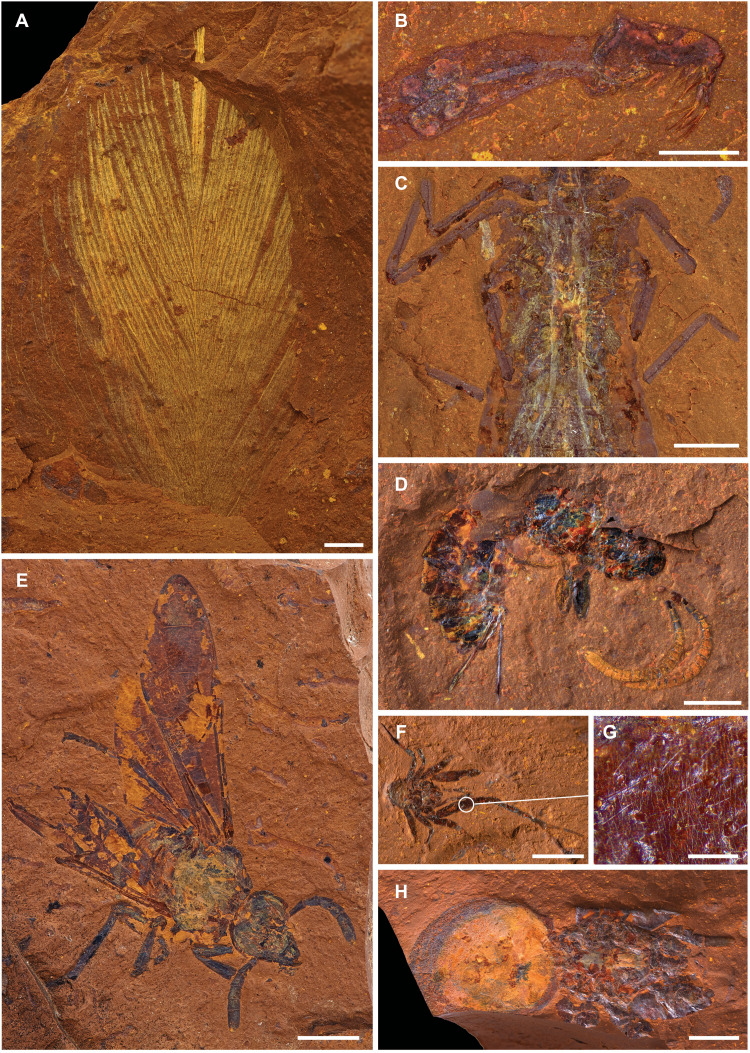
Animal fossils. (**A**) Feather (AM F.145096); (**B**) larvae of phantom midge (*Chaoborus* sp.: Chaoboridae, Diptera) (AM F.146585); (**C**) dragonfly naiad (AM F.145848); (**D**) parasitoid wasp (AM F.146703); (**E**) sawfly (Tenthredinoidea: Symphyta) (AM F.145093); (**F**) tangle web spider (Theridiidae) (AM F.145550); (**G**) close-up of setae on spider (AM F.145550); and (**H**) mygalomorph spider (Mygalomorphae) (AM F.146659). Scale bars, 100 μm (G), 750 μm (B), 1 mm (A and D), 2 mm (E), 2.5 mm (C and F), and 5 mm (H).

**Fig. 5.**
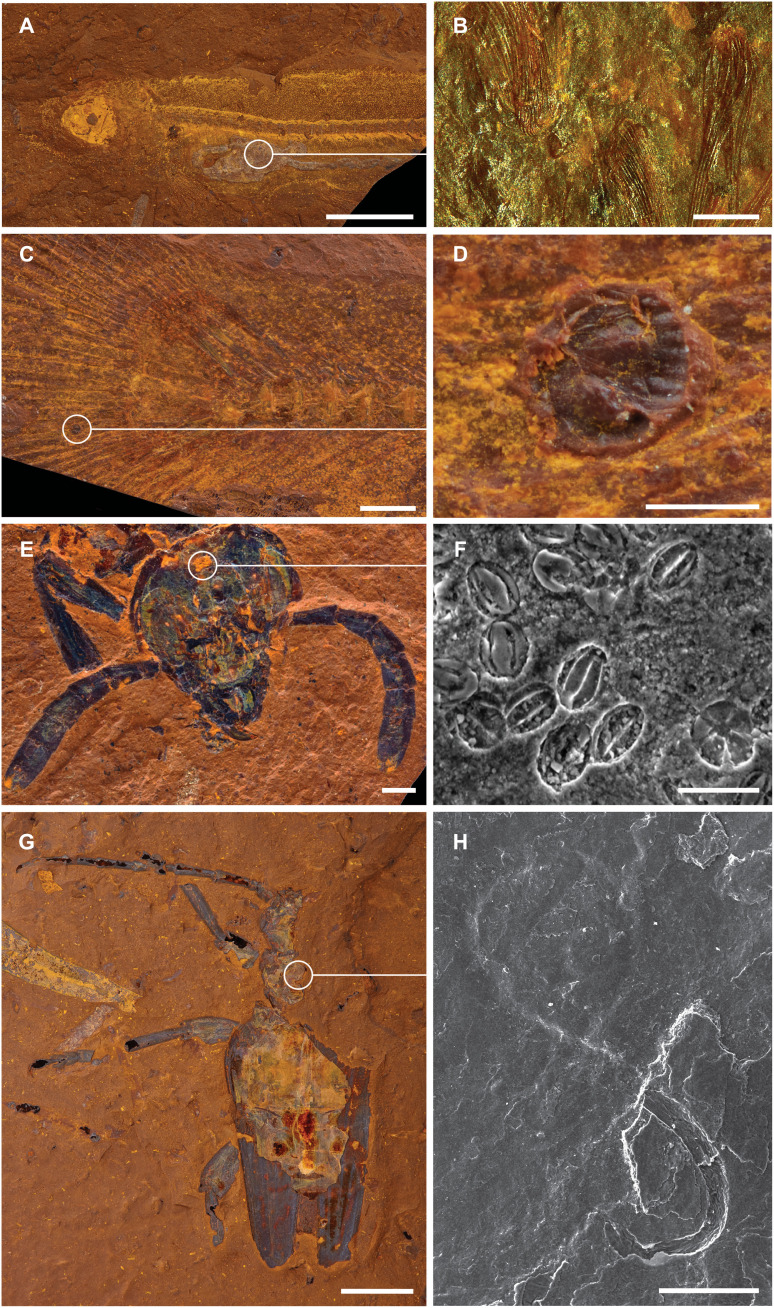
Evidence of biotic interactions. (**A** and **B**) Stomach contents of fish (AM F.145094) showing that it fed predominantly on phantom midges (*Chaoborus* sp.); (**C** and **D**) a parasitic glochidium attached to the caudal fin of a fish (AM F.146607); (**E** and **F**) pollen preserved on the head of a sawfly (AM F.145093); and (**G** and **H**) a phoretic nematode attached to the body of a longhorn beetle (Cerambycidae: Coleoptera; AM F.145098). Scale bars, 25 μm (F), 100 μm (H), 250 μm (B and D), 1 mm (C and E), 5 mm (G), and 10 mm (A).

**Fig. 6.**
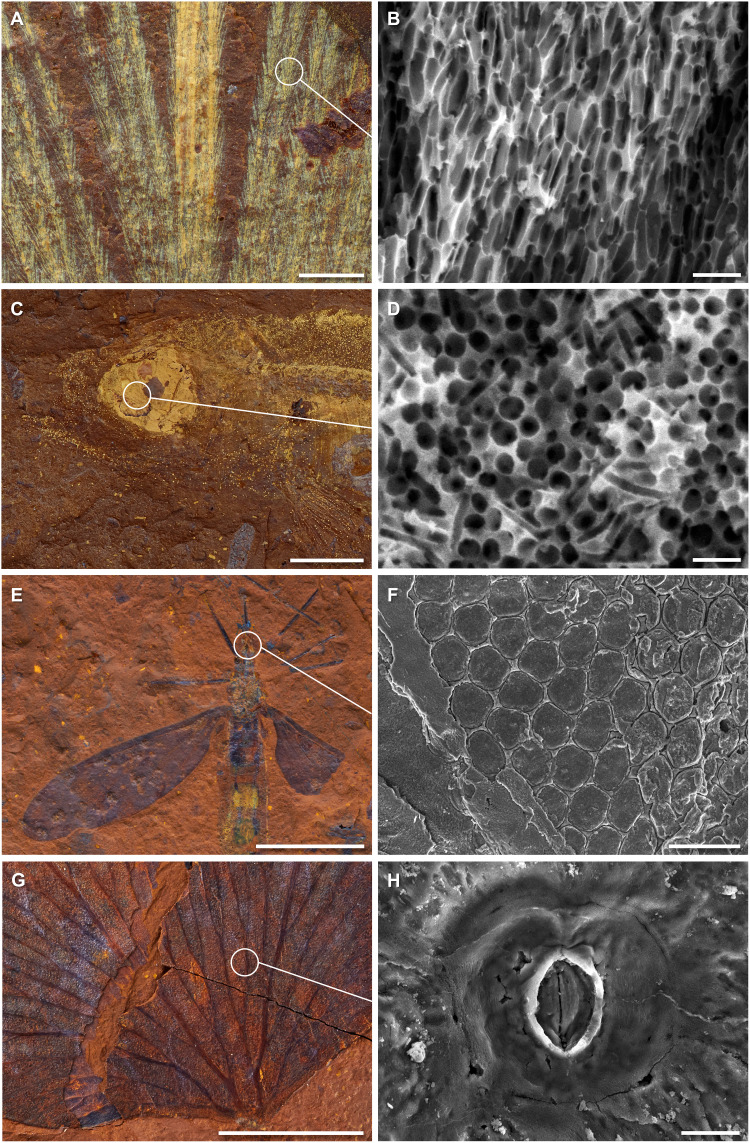
High-fidelity preservation in fossils from McGraths Flat. (**A** and **B**) Microstructure of fossil feather (AM F.145096) showing melanosome preservation; (**C** and **D**) melanosomes preserved in the eye of a fossil fish (AM F.145094); (**E** and **F**) crane fly (Limoniidae cf. *Tonnoirella*) fossil (AM F.146583) with ommatidia of the eye preserved; (**G** and **H**) *Lygodium* leaf (AM F.146600) exhibiting the structure of the stomata. Scale bars, 1 μm (B and D), 10 μm (H), 50 μm (F), 0.5 mm (A), and 5 mm (C, E, and G).

McGraths Flat preserves a wide diversity of fossilized insects and arachnids (Fig. 4 and table S4). Merolimnic (those with an aquatic life stage) insect larvae are very common; in some cases, more than a dozen specimens have been found on a 25-cm^2^ surface. Larvae and pupae of phantom midges (*Chaoborus* sp.: Chaoboridae, Diptera) are, by far, the most numerous fossil insects (Fig. 4B), although larvae of nonbiting midges (Chironomidae, Diptera), caddisflies (Trichoptera) and alderflies (Sialidae, Megaloptera), and naiads of dragonflies (Odonata; Fig. 4C) and mayflies (Ephemeroptera) are also abundant. Terrestrial insect taxa from McGraths Flat include assassin bugs (Reduviidae, Heteroptera), weevils (Curculionoidea, Coleoptera), a water beetle (Dytiscidae, Coleoptera), a longhorn beetle (Cerambycidae, Coleoptera) (Fig. 5G), four cicadas (Cicadoidea, Hemiptera), a froghopper (Cercopoidea, Hemiptera), two termite wings (Mastotermitidae, Isoptera), ants (Formicidae), parasitoid wasps (Fig. 4D) as well as a near-complete fossil of a sawfly (Hymenoptera) (Fig. 4E), and craneflies (Limoniidae, Diptera) (Fig. 6, E and F). In addition, 13 spiders have been found (Fig. 4, F and H), all of which preserve fine morphological features, such as individual setae on the limbs and spiracular valves on the body (Fig. 4G). Isolated lepidopteran wing scales preserve structural details such as ridges and cross ribs (Fig. 3, E and F). Many of the fossil arthropods represent undescribed genera and are the first Miocene records of their families in Australia.

Vertebrate remains are relatively uncommon in the deposit; however, a single feather (Fig. 4A) and more than a dozen specimens of fish have been collected (Fig. 5, A and C). The feather has a straight rachis and symmetrical vanes with well-preserved barbs and barbules (Fig. 6A). On the basis of its morphology and its pennaceous aspect, the specimen is likely a contour feather of a bird similar in size to a modern sparrow. All but one of the fish found to date appear to represent the same taxon (cf. Retropinnidae). The other cannot be identified beyond Perciformes. In some fish specimens, the gastrointestinal tract along with the stomach content is preserved.

### Palaeoclimatic reconstruction

**Fig. 7.**
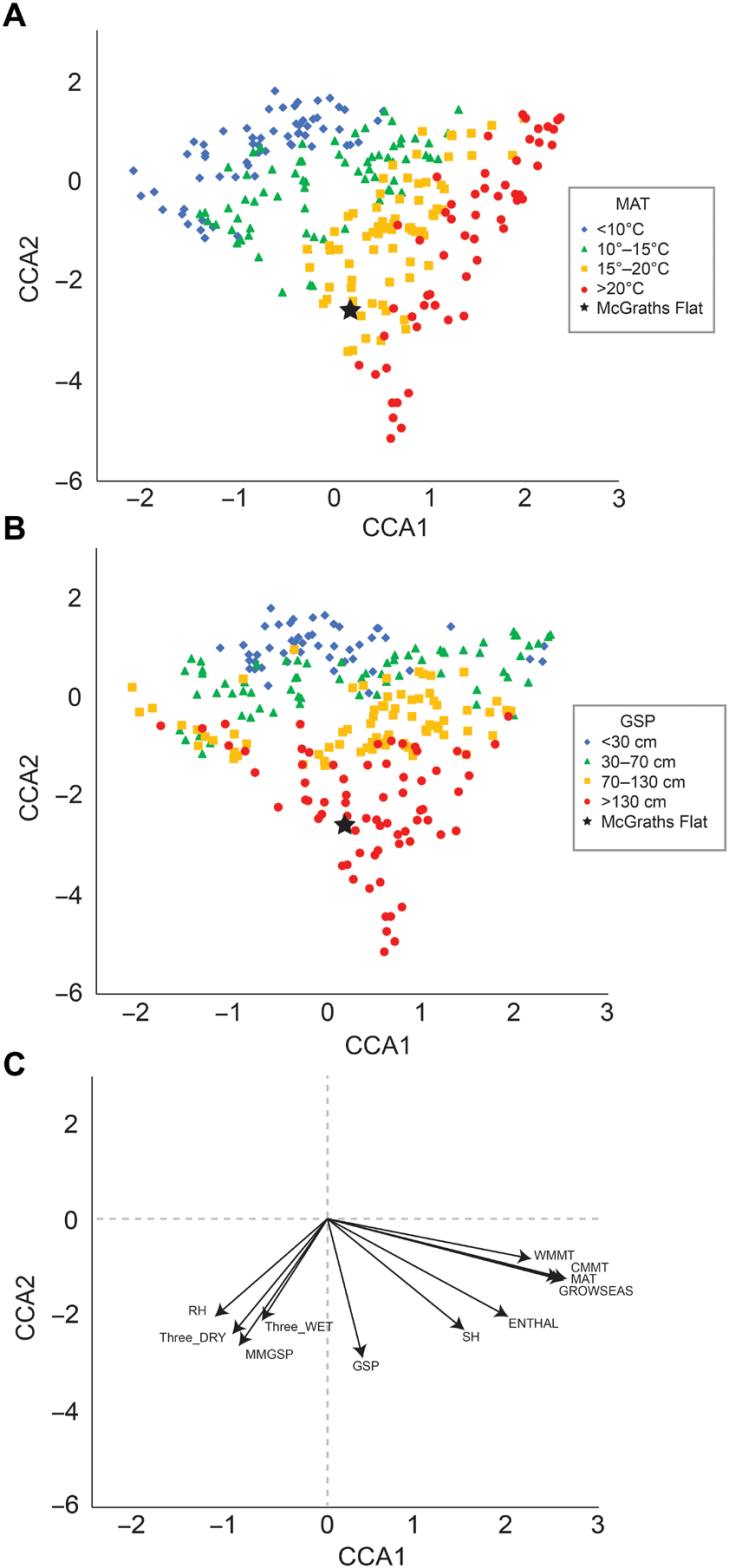
CLAMP results. (**A**) Axis 1 versus axis 2 canonical correlation analysis (CCA) biplot of modern flora localities overlaid with the mean annual temperature (MAT) data. (**B**) Axis 1 versus axis 2 CCA biplot of modern flora localities overlaid with growing season precipitation (GSP) data. (**C**) The environmental vectors in axis 1 versus axis 2 space; vectors: WMMT, warm month mean temperature; CMMT, cold month mean temperature; GROWSEAS, length of the growing season; MMGSP, mean monthly growing season precipitation; Three_WET, precipitation during the three consecutive wettest months; Three_DRY, precipitation during the three consecutive driest months; RH, mean relative humidity; SH, specific humidity; ENTHAL, enthalpy. McGraths Flat is depicted with a star.

We estimated the local palaeoclimatic conditions at the site using the Climate Leaf Analysis Multivariate Program (CLAMP) (*17*, *18*). A dataset of leaf characters was compiled from the macroflora collection of the Australian Museum (table S5). This dataset had a completeness index of 49%, which is sufficient to provide a preliminary estimate of the climate at the time of deposition. The analysis estimated a mean annual temperature of 17.0°C, a warm month mean temperature of 26.4°C, and a cold month mean temperature of 7.0°C. The growing season was estimated to be 9.3 months, with a growing season precipitation of 2330 mm, 962 mm/month during the three wettest months of the year, and 254 mm/month during the three driest months of the year (Fig. 7).

### Quality of preservation

The fossils from McGraths Flat frequently exhibit a high degree of soft tissue preservation that is close to, or comparable to, other Konservat-Lagerstätten such as the Crato Formation, Lake Eckfeld, and Libros (*19*, *20*). When fossiliferous rocks from McGraths Flat are processed using a hammer and chisel, most fossils split through the body cavity, rather than exposing the external surface of the body, revealing the internal organs and tissues. For example, the spiders from the deposit show internal details of the trichobothrium (Fig. 4C), and some insects exhibit preservation of the tracheal system. In some instances, particularly in alderfly larvae and dragonfly naiads, digestive and muscular systems are also preserved (Fig. 4C). In other insect fossils, the microstructure of compound eyes is clearly discernible, such as the multiple ommatidia in the eye of a fossil cranefly (Limoniidae) (Fig. 6, E and F).

Vertebrate remains also show highly detailed preservation. For example, the feather preserves elongate, aligned, and densely packed melanosomes, similar to those seen in dark or iridescent feathers (Fig. 6, A and B) (*21*, *22*). Likewise, fish fossils show melanosomes in the tissues of the eye (Fig. 6, C and D) and in melanophores of the skin, which may allow reconstruction of color patterns. The size, shape, and location of the melanosomes confirm their identity (e.g., in the fish, melanosomes are observed within the outline of individual melanophores).

The detailed preservation extends to the plant material. Delicate fern fronds rarely fossilize well, but, within this deposit, they often show stomial cells of sporangia and details of stomata (Fig. 6, G and H). Most unusual is the preservation of the spores and pollen that are found as molds rather than organic remains (Fig. 3, H and I). For instance, *Nothofagidites* pollen grains show pits rather than the usual fine spinose sculpture (fig. S5). In rare cases, both molds and casts are found, as exemplified by a spore of *Rugulatisporites trophus* (Fig. 3, H and I), in which the imprint of the mold of the outer exine is apparent and surrounds a cast of the intine (cellulose wall around the protoplasm). This form of preservation prevents retrieval by standard palynological extraction methods (e.g., acid digestion). The detailed preservation of microfossils in this deposit has implications for palynological studies of other goethite deposits, as it demonstrates that scanning electron microscopy (SEM) scanning of oxidized, iron-rich sediments (previously deemed unproductive using standard palynological techniques) can, under certain circumstances, enable the identification of microfossils and aid in the dating of sites.

## DISCUSSION

The northward movement of Australia during the Cenozoic, combined with global climate variations, has left an indelible signature on the extant vegetation. Rainforest communities that covered much of the continent in the Eocene contracted as Australia became drier (*2*, *23*). Today, except in the Monsoon Tropical Zone, rainforests are largely confined to areas east of the Great Dividing Range where they occur in pockets near the east coast, often in fire-protected parts of the landscape. The Cenozoic record of these changes is limited by scattered floras that are usually poorly age constrained, and few regions contain continuous records of sedimentation and associated floristic information. Our findings provide evidence that the vegetation that persisted west of the Great Dividing Range into the mid-Miocene included mesic rainforest elements.

CLAMP reconstructs the climate as reasonably warm and wet. Within the Southern Hemisphere CLAMP calibration dataset (*24*), localities with similar precipitation measures (mean monthly growing season precipitation, mean dry month precipitation, and mean wet month precipitation) include Conondale National Park, Bulburin National Park, and Bowling Green Bay National Park, all of which are found close to the eastern coast of Queensland and contain large areas of “remnant” subtropical rainforest. In contrast, localities with similar temperature characteristics (mean annual temperature, wet month mean temperature, and cold month mean temperature) include Banks Peninsula, New Zealand (lowland podocarp forest), and Karee Kranz, South Africa (woodland). Australia has been in a phase of northward drift since the Eocene. At the time of deposition, McGraths Flat would have been located at somewhere between 37°S and 40°S [estimated using Gplates (*25*)] compared with 32.2°S today. Our analysis suggests that these latitudes had sufficient rainfall and temperature in the Middle Miocene to facilitate the persistence of pockets of rainforest.

A lack of fossil sites that preserve insects and arachnids has made it difficult to determine the invertebrate faunal composition of Miocene rainforests in Australia and infer their responses to the changing environment. This report provides an important comparison for other Miocene fossils sites, such as the Zhangpu Konservat-Lagerstätte, China (*26*) and Foulden Maar, New Zealand (*27*), and shows that the Miocene rainforests of central Australia hosted a diverse array of plants, insects, and spiders. The flora and fauna found at McGraths Flat are in stark contrast to those found in the area today. The dry sclerophyll forest currently found around Gulgong is primarily composed of taxa adapted to dry conditions such as *Eucalyptus* and *Acacia*. The palaeoenvironment is far more diverse, containing many taxa that, today, are only found in wetter environments such as *Nothofagus* and *Lygodium*. The environment of the site supports hypotheses, previously formed through the study of microfossils and molecular phylogenies, that shifts in climate since the Miocene substantially reshaped the faunal and floral composition of the Australian landscape (*3*, *4*).

**Fig. 8.**
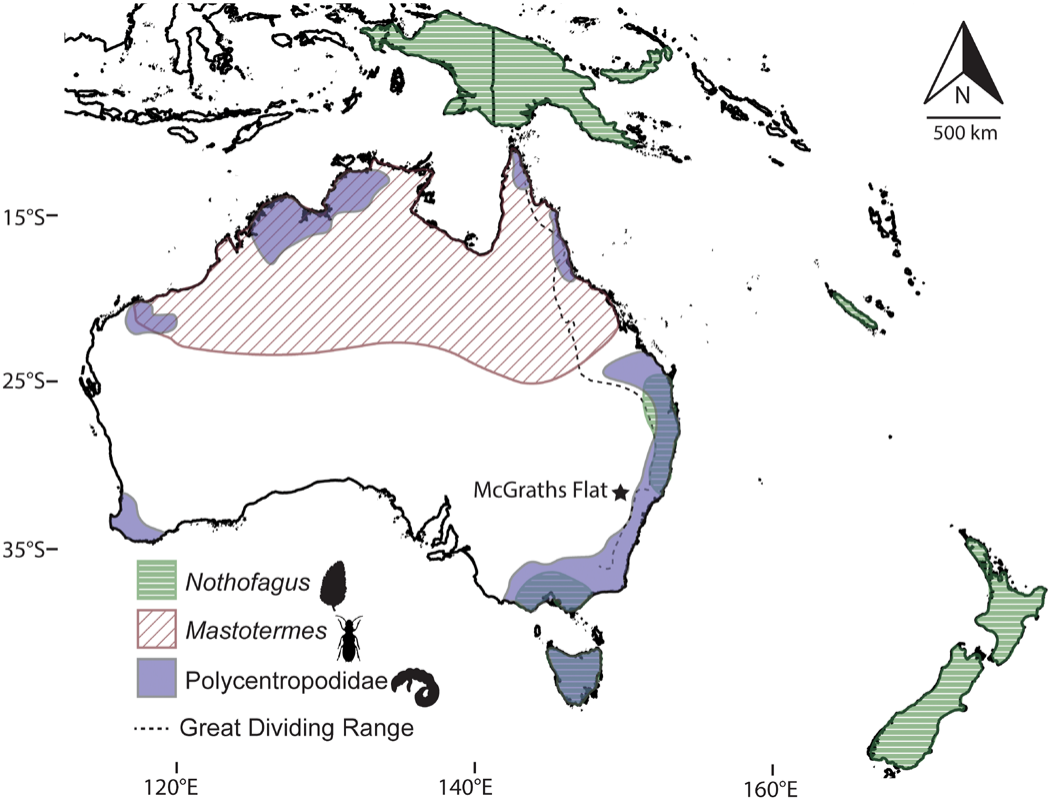
Shifts in the geographic range of selected plant and animal taxa since the Miocene. The current geographical ranges of *Nothofagus* (southern beeches), *Mastotermes* (giant termites), and Polycentropodidae (trumpet-net and tube-making caddisflies) are indicated by colored and shaded regions. The location of the fossil site is depicted with a star.

Many insects from McGraths Flats have close relatives that are now restricted to small patches of rainforests in the northern and eastern parts of the continent: (i) The presence of two different mastotermitid species, which are now represented by a single species (*Mastotermes darwiniensis*) (Fig. 8), suggests that this group was distributed further south during the hotter and wetter conditions of the Miocene and has since contracted or shifted its range to the north of the continent where these climatic conditions still exist today; (ii) many alderfly (Megaloptera) fossils are found at McGraths Flat; in extant ecosystems, these taxa are restricted to the wet tropic and mesic forests of eastern Australia (*28*); and (iii) the caddisfly family Polycentropodidae now occurs in patches close to the coast throughout Australia. Within New South Wales, they are restricted to the east of the Great Dividing Range where precipitation levels are greater (Fig. 8). The fact that so many Miocene insect groups are now only found in mesic rainforests suggests that insects responded to aridification by moving to areas with suitable living conditions rather than adapting to a drier environment.

The diverse fossil assemblage at McGraths Flat, combined with high-fidelity preservation, allows for detailed investigation of species interactions. Predator-prey relationships are recorded as stomach contents. For example, some of the fossil fish show a well-preserved gastrointestinal tract that contain the remains of numerous chaoborid larvae (their prehensile labral bristles seem especially resistant to digestion; Fig. 5, A and B) and, in one case, a partially digested dragonfly wing. Nonchaoborid insect remains are rare, which suggests that the fish consumed mostly chaoborids, the most available prey in the environment before deposition. Interactions between insects and plants are documented from leaf damage. In addition, dozens of pollen grains were found on the head of a sawfly, which points to a pollinator-plant interaction (Fig. 5, E and F). Commensalistic relationships have also been observed, for instance, the preservation of a single nematode beneath the pronotum of a cerambycid beetle suggests a phoretic relationship between these taxa (Fig. 5, G and H), and the glochidium of a freshwater mussel attached to the caudal fin of one of the fossil fish (Fig. 5, C and D) documents a parasitic relationship. While glochidia are not unusual components of freshwater fossil deposits, this is the first example of a glochidium attached to a host in the fossil record. These examples highlight how the well-preserved fossils from McGrath Flat enhance our understanding of the biology and ecology of the Miocene rainforest biota of Australia.

## MATERIALS AND METHODS

### Specimen collection

Specimens were recovered during seven short trips (3 to 4 days each) to the site. Initial fossil collection focused on fossils that had been brought to the surface through tilling. After mapping the surface geology, an excavator was used to create seven pits to clarify the stratigraphic relationships between the rock units. A further six pits were dug to excavate rocks from the fossiliferous layer in situ. All fossil specimens are deposited in the paleontology collection of the Australian Museum in Sydney, New South Wales (accession prefix AM F).

### Imaging

Conventional (light) photographs were taken with either a Canon EOS 7D Mark II camera mounted on a BK Lab Plus Lab imaging system (Dun Inc., Charlottesville, VA, USA), a Leica DVM6 microscope (Leica Microsystems GmbH, Wetzlar, Germany), or a Nikon D100 digital camera using a standard copy stand. SEM images were taken with a FEI Quanta 650F variable-pressure field-emission SEM (FEI Company, Hillsboro, OR). The FEI microscope was operated at 15 kV, with a spot size of 2.45 and working distances of about 12 to 15 mm. Notably, the high conductivity of the rocks from McGraths Flat allowed high-resolution SEM imaging without any coating. All microfossils were imaged in situ (i.e., in the matrix) rather than using acid preparation. Micro x-ray fluorescence (μXRF) was undertaken by directly sampling across the surface of a rock containing a fish spine (AM F.146602) using a benchtop M4 Tornado μXRF (Bruker Corp., Billerica, MA). This allowed us to sample both the fossil and matrix for differences in chemistry. The instrument consists of a Rh anode metal-ceramic x-ray tube. The measurement was carried out under vacuum condition (20 mbar). The following parameters were used for elemental mapping analysis: x-ray tube, 45 kV and 600 μA; area mapping point distance, 60 μm; time/pixel, 600 ms; 1 cycle; maximum energy, 40 keV; and maximum pulse throughput, 130 kilo counts per second.

### CLAMP analysis

CLAMP analysis uses established relationships between leaf form and environmental conditions to predict the climatic conditions of paleontological communities. We compiled a dataset of 31 physiognomic parameters from the flora present at McGraths Flat. The dataset yielded a completeness index of 49%, mainly owing to missing leaf apices and leaf base characteristics. We compared the site to the calibration dataset of 90 Southern Hemisphere localities presented in (*24*) and the Physg3arcAZ Calibration dataset, which consists of 173 modern vegetation sites, predominantly from the Northern Hemisphere (*9*).

## Notes

View/request a protocol for this paper from *Bio-protocol*.
