# Supplementary material for: A Lagerstätte from Australia provides insight into the nature of Miocene mesic ecosystems
Source: Sci Adv. 2022 Jan 7;8(1):eabm1406. doi: 10.1126/sciadv.abm1406 (PMC8741189; doi:10.1126/sciadv.abm1406)
Supplement: Supplementary file 1 — Supplementary Methods Figs. S1 to S6 Tables S1, S3, S4, and S5 [file sciadv.abm1406_sm.pdf]

Science Advances
AAAS

Supplementary Materials for

A Lagerstätte from Australia provides insight into the nature of Miocene mesic ecosystems

Matthew R. McCurry*, David J. Cantrill, Patrick M. Smith, Robert Beattie, Mary Dettmann, Viktor Baranov, Charles Magee, Jacqueline M. T. Nguyen, Marnie A. Forster, Jack Hinde, Ross Pogson, Helen Wang, Christopher E. Marjo, Paulo Vasconcelos, Michael Frese*

*Corresponding author. Email: matthew.mccurry@austmus.gov.au (M.R.M); michael.frese@canberra.edu.au (M.F.)

Published 7 January 2022, *Sci. Adv.* **8**, eabm1406 (2022)
DOI: 10.1126/sciadv.abm1406

The PDF file includes:

Supplementary Methods
Figs. S1 to S6
Tables S1, S3, S4, and S5

Other supplementary material for this manuscript includes the following:

Table S2

Supplementary methods

XRD analysis

Three samples of matrix and fossil from McGraths Flat (Table S1) were analyzed using a Panalytical X'Pert Pro X-ray diffractometer with vertical theta-theta goniometer, proportional counter, graphite monochromator, and copper anode X-ray source. Scans were performed at 45 kV and 40 mA, with a 0.5-degree divergence slit and 1-degree anti-scatter slit, counting for 1 second per 0.02-degree step from 5 to 75 degrees of two-theta. Scans were processed with Panalytical HighScore Plus software and peak patterns were matched with the PDF-4/Minerals 2019 database. Peak matches indicated almost pure goethite, FeO(OH), with a small amount of quartz in some samples. Some goethite peak matches were possibly closer to a slightly aluminous goethite, but additional chemical data are required to confirm the presence of aluminium.

$^{40}Ar/^{39}Ar$ dating

Flux monitor: GA 1550 @ 98.5 ±0.8 Ma (*29*).

$(^{36}Ar/^{37}Ar)_{Ca}$ correction factor	1.289^{-4}
$(^{39}Ar/^{37}Ar)_{Ca}$ correction factor	8.281^{-4}
$(^{40}Ar/^{39}Ar)_{K}$ correction factor	8.342^{-2}
$(^{38}Ar/^{39}Ar)_{K}$ correction factor	1.172^{-2}
$(^{38}Ar)_{Cl}/(^{39}Ar)_{K}$ correction factor	8.065^{-2}
Ca/K conversion factor	1.9
Lambda ^{40}K	5.543^{-10}

Mineral separation and characterization

Samples were crushed and separated at the Research School of Earth Science, The Australian National University, Canberra, Australia, using normal mineral separation techniques. The most pristine sections of the basalt were cut from the rock, crushed, and sieved to 250-µm grain size, de-slimed, dried, and washed. Grains were again cleaned in de-ionized water and wrapped in aluminum foil in preparation for irradiation.

Sample irradiation details

Aluminum-wrapped sample packets were placed into a quartz irradiation canister together with aliquots of the flux monitor GA1550 (*29*). The GA1550 standards are dispersed

throughout the irradiated cannister, between the unknown age samples. Approximately 30 of the GA1550 standards were analyzed, a linear best fit method was then used for the calculation of the J-factor and J-factor uncertainty. Packets containing K_2SO_4 and CaF_2 were placed in the middle of the canister to monitor ^{40}Ar production from potassium and calculate necessary correction factors.

Irradiation of samples for ^{40}Ar/^{39}Ar analysis was undertaken at the University of California Davis' McClellan Nuclear Research Center, CA, USA, prior to analysis. Irradiated samples were unwrapped on their return to the Australian National University, and then rewrapped in tinfoil ready for analysis.

^{40}Ar/^{39}Ar procedures:

Samples and standards were analyzed in the Argon Laboratory at the Research School of Earth Science, The Australian National University, Canberra, Australia.

A furnace step-heating technique was used to extract argon from the samples to ensure 100% release of ^{39}Ar (*30*), while the flux monitors crystals (GA1550 biotite) were fused using a CO_2 continuous-wave laser; gases extracted from both samples and standards were analyzed in the Argus VI mass spectrometer. Samples were analyzed in 30 steps and with temperatures of the overall schedule rising from 450 to 1450 °C (*31*). Backgrounds were measured prior to each step analysis and subtracted from each step analysis. The furnace cleaning procedure entailed heating and degassing four times at 1450 °C for 15 minutes and the gas pumped away prior to the loading of the subsequent sample. Gas released from flux monitors and each step of sample analyses was exposed to three different Zr-Al getters to remove active gases for 10 minutes, and the purified gas was then isotopically analyzed in the mass spectrometer. ^{40}K abundances and decay constants are those of Steiger and Jäger (1977) (*32*). Stated precisions for ^{40}Ar/^{39}Ar ages include all uncertainties in the measurement of isotope ratios and are quoted at the one sigma level and exclude errors in the age of the fluence monitor GA1550 (98.5 ±0.8 Ma) (*29*). The reported data have been corrected for system backgrounds, mass discrimination, fluence gradients, and atmospheric contamination. The ^{40}Ar/^{39}Ar dating technique is described in detail by MacDougall and Harrison (1999) (*33*) and adapted as in Forster and Lister (2009) (*30*).

^{40}Ar/^{39}Ar sample information

Sample name: 435839350

J-factor: 1.1815E-3

± % J: 0.2419

Mineral: basalt (whole rock)

Measurement date: 17/01/2020

The background levels within the mass spectrometer were measured prior to every step of the analysis and subtracted from the isotope intensities for ^{40}Ar, ^{39}Ar, ^{38}Ar, ^{37}Ar, and ^{36}Ar. The interfering nuclear reactions and correction factor ratios for the isotopes listed above were calculated. These are measured for the reactions and uncertainties of $(^{40}Ar/^{39}Ar)_K$, $(^{39}Ar/^{37}Ar)_{Ca}$, $(^{36}Ar/^{37}Ar)_K$, $(^{38}Ar/^{39}Ar)_K$, and $(^{38}Ar)_{Cl}/(^{39}Ar)_K$, and were calculated prior to sample analysis.

Data reduction was done with an adapted version of Noble Software (written and adapted by the Australian National University Argon Laboratory). The data reduction was based on optimizing MSWD (the mean square of weighted deviates) of isotope intensities with an exponential best fit methodology. The discrimination factor was calculated by analyzing Air Shots analysis on either side of sample analysis, based on the $^{40}Ar/^{36}Ar$ ratio (298.57), and the calculation of the 1 amu was used for the discrimination factor.

$^{40}Ar/^{39}Ar$ results are supplied in Supplemental Table S2.

Detrital zircon analysis

Three rock samples were received by Geoscience Australia and assigned GA numbers (3081571, 3081572, and 3081573) before being processed for the isolation of zircons. Samples 3081571 and 3081572 were fossiliferous limonitic fine-grained sandstone representing sandy layers at the base of the fossil deposit. Sample 3081573 was an iron-rich quartz pebble conglomerate representing the lag deposit found bellow the fossil deposit. Samples 3081571 and 3081572 yielded typically rounded and equant detrital zircons, and a sub-population of large, relatively unrounded elongated grains. These elongated grains were mounted alongside the unpicked separates.

All samples were analyzed on a SHRIMP IIe using standard U-Th-Pb methodology (*34*). Samples were normalized to a Temora 2 zircon with an age of 416.8 Ma (*35*). The error of the mean of 61 Temora 2 analyses was 0.1%. An excess spot to spot error of 0.5% was sufficient to explain all dispersion in the reference material data.

Sample 3081571 had 108 detrital zircon analyses, including 14 analyses of elongated grains. Sample 3081572 had 105 analyses, including 13 of elongated grains. Sample 3081573 had 109 zircons analyzed. Of these 108 grains analyzed for sample GA3081571, 11 were discordant beyond the uncertainty envelope. The remaining 97 zircon ages ranged from Cretaceous to Paleoarchean. The youngest three zircons ranged from 133 to 147 Ma, but do not form a single group within uncertainty of each other.

The most common age in GA3081571—with just under half the zircons—is in the Cambrian to Ediacaran "Pacific Gondwanan" population related to the assembly of Gondwana (*36*). These peaks are at 490 Ma, 525 Ma, and ~560 Ma. Another prominent peak is at 430 Ma, the age of widespread S-type plutonism and volcanism in New South Wales. There are also several ~180 to 230-Ma, ~950 to 1150-Ma, and 640 to 680-Ma grains, with scattered older Paleoproterozoic and Archean grains.

Sample GA3081572 had 105 analyses including 13 of the elongated grains. Of these, eight were discordant, leaving a sample of 97 grains. Their ages were broadly similar to sample GA3081571, with ages ranging from Jurassic to Neoarchean. The youngest three zircons ranged from 189 to 215 Ma, but do not form a single group within uncertainty of each other.

The most common age of GA3081572 is Pacific Gondwanan, with peaks at 522 Ma and a broad 550 to 565-Ma peak. These grains account for just under half of the total zircons. There are smaller populations at 360 and 430 Ma, and scattered grains from 800 to 1050 and 180 to 300 Ma. There is only one Archean grain, and only four grains from the Paleoproterozoic.

One hundred and nine zircons were analyzed from sample GA3081573. Eighteen of these analyses were discordant. Of the remaining 91 grains, the youngest three are not within error of each other, and range from 220 to 243 Ma. The age distribution of the Paleozoic and Proterozoic grains is quite different to the other two samples, however. The most prominent peaks are ~430 Ma and ~390 Ma, and these account for just over a third of the zircons. Pan-African zircons were present but made up only one quarter of the total zircon population. The main peaks are at 485 Ma, 515 Ma, and 560 Ma. There are also broad distributions in the 220 to 320 and 880 to 1180 Ma range. There are four Paleoproterozoic and three Neoarchean grains.

As samples 3081571 and 3081572 contain Miocene fossils, the zircon data give no meaningful constrains on the depositional age. For sample GA3081573 the deposition age must be younger than the Triassic youngest zircons in this sediment, as discussed below. The interpretation of the Permian-to-Mesozoic zircon data is difficult. Detrital zircon age interpretation generally relies on the population statistics of the zircons. However, with the exception of the three ~180-Ma zircons in sample GA3081571, none of the zircons younger than ~330 Ma form groups of three or more zircons. This is unusual; however, as no other Triassic samples were analyzed in months prior to this analysis, the result is not likely a consequence of sample contamination.

Due to the young age of these zircons, it is possible that this spread is caused by variable Pb loss subparallel to concordia, as the ^{207}Pb precision may not be sufficient to distinguish this sort of Pb loss in the Mesozoic. This is unlikely for samples GA3081571 and GA3081572. In sample GA3081571, all of the grains younger than 300 Ma are low in uranium (<500 ppm) and have less than 1% common ^{206}Pb, so are unlikely to have been disturbed. In sample GA3081572, the U contents are up to 1100 ppm, but the same is generally the case. This cannot be said for sample GA3081573.

The youngest grain in sample GA3081573 has more than 2% common ^{206}Pb, and the U content is well over 1000 ppm. So, it is conceivable that this zircon is as old or older than the 236 Ma next-youngest concordant grain and has lost Pb to appear younger. Thus, the maximum depositional age for this sample is approximately 236 Ma, based on two zircon grains that are within uncertainty of each other. The presence of an older well-behaved Triassic grain further supports the interpretation that the matrix is Mesozoic (or younger) sediment. The results of this analysis are presented in Fig. S4.

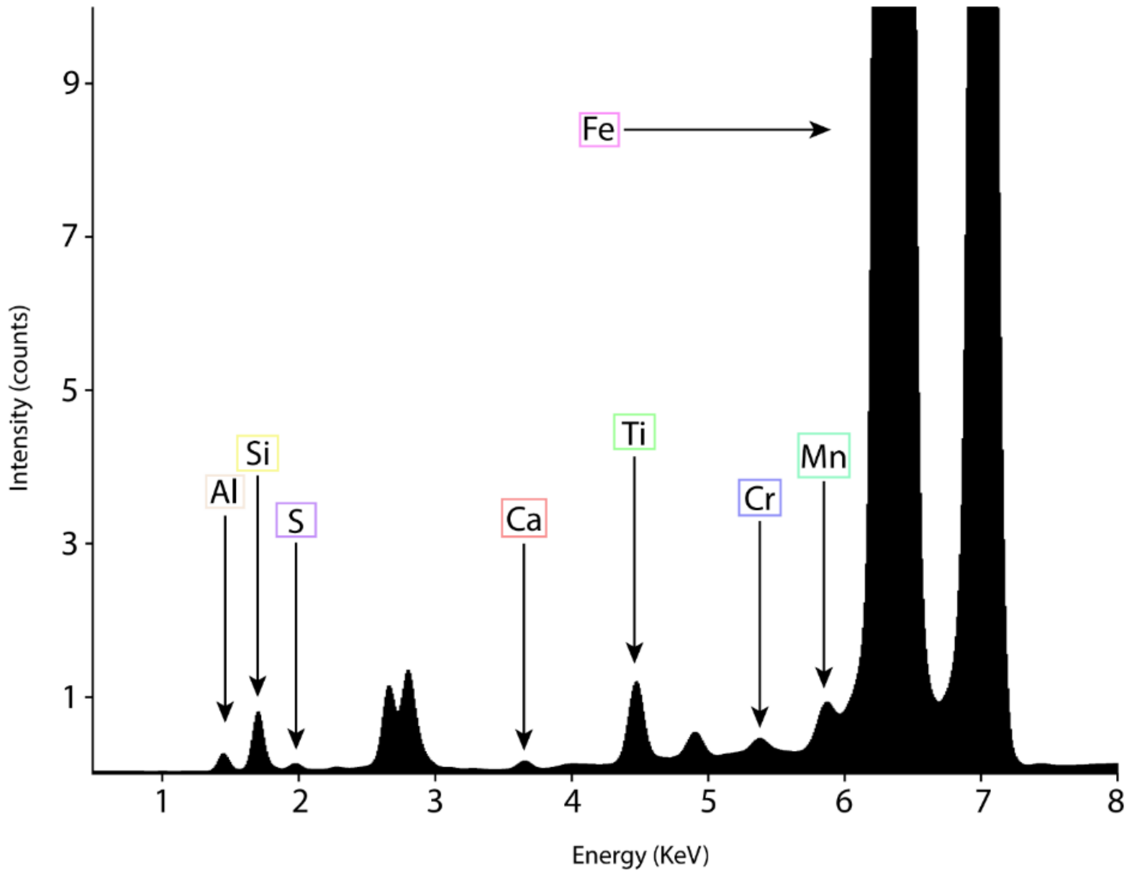

Fig. S1. XRF results. Graph of XRF spectra from ta sample of fossil and matrix from McGraths Flat. The matrix and fossil primarily contain iron hydroxides, with small concentrations of Mn, Cr, Ti, S, Si, and Al.

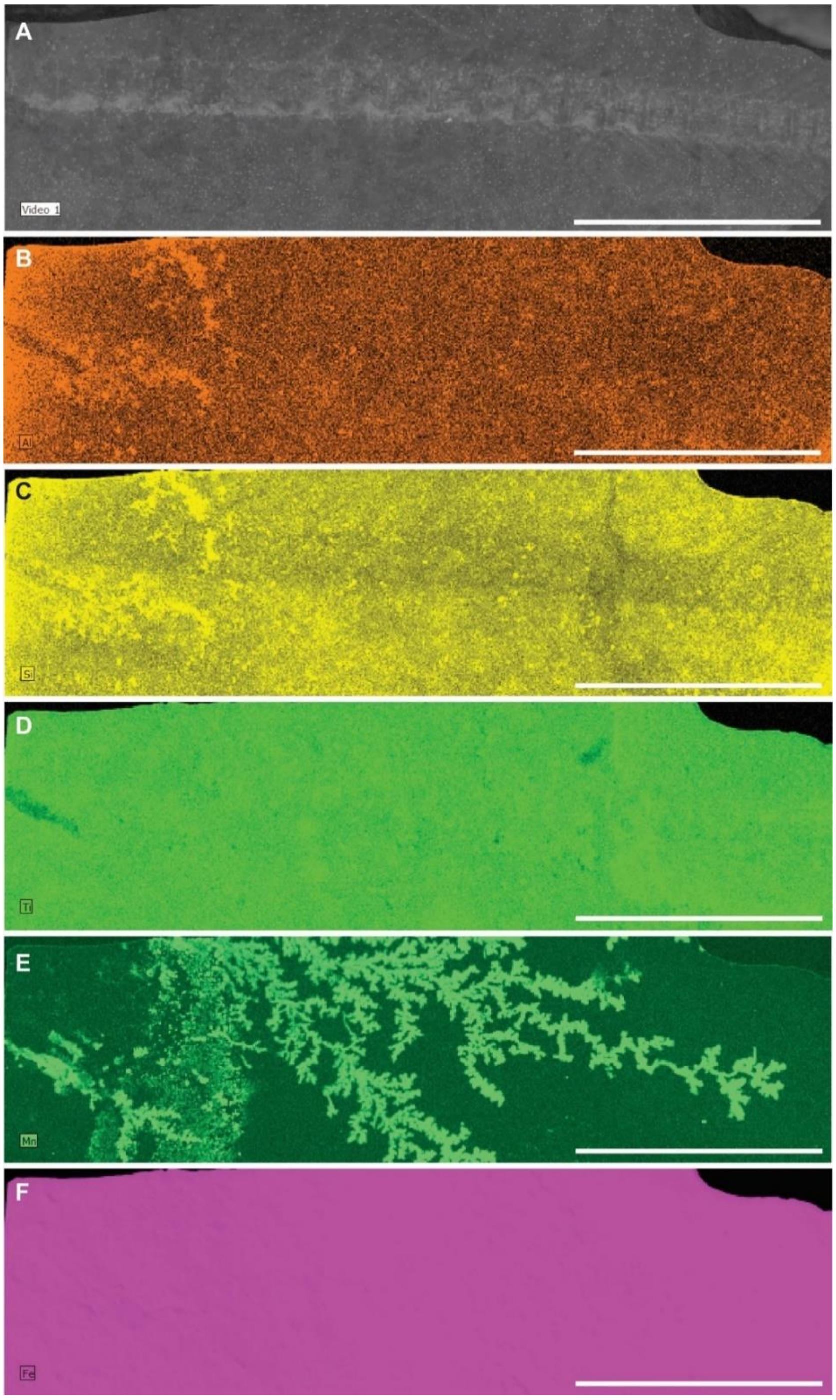

Fig. S2. XRF images. (**A**) black and white image of the fossil; (**B**) distribution of aluminum (Al); (**C**) distribution of silicon (Si); (**D**) distribution of titanium (Ti); (**E**) distribution of manganese (Mn); and (**F**) distribution of iron (Fe). Scale bars, 10 mm.

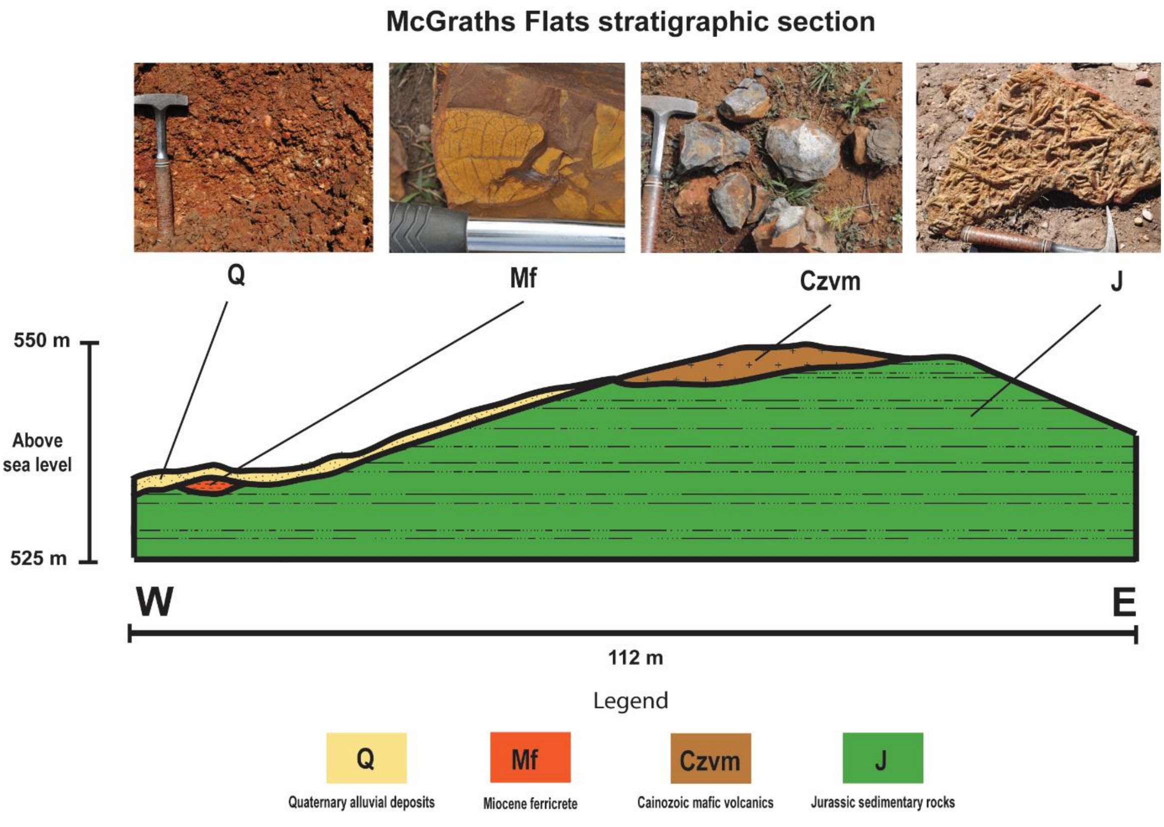

Fig. S3. Stratigraphic cross section at McGraths Flat. Stratigraphic East–West cross-section through the McGraths Flat site. The position of the McGraths Flat bed (Mf) is depicted in relation to surrounding stratigraphic units. Field photographs are provided to show examples of the rock types and units discussed. Scale of photographs is provided by geological hammers.

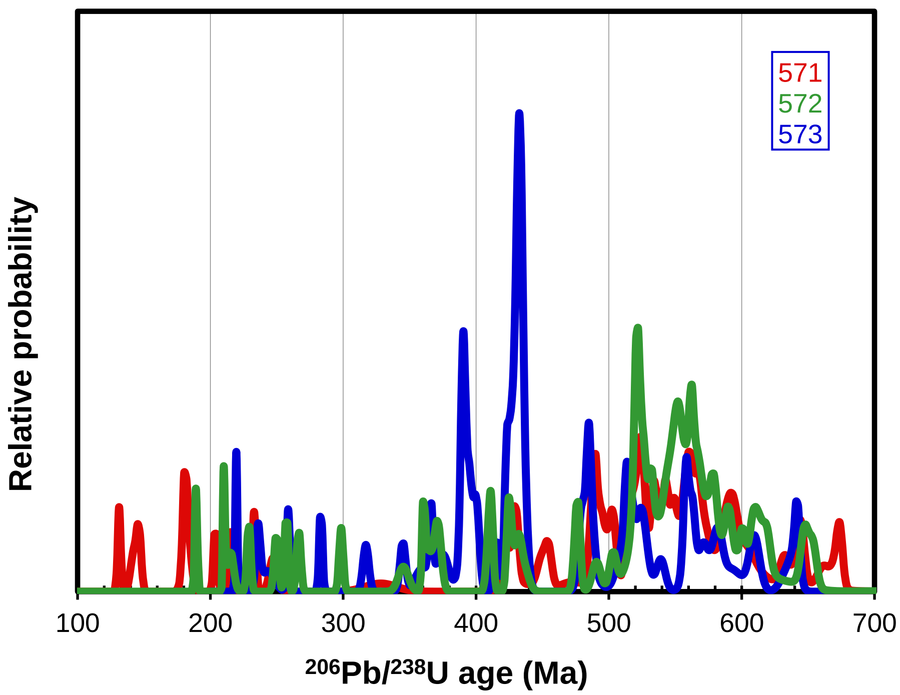

Fig. S4. Zircon dating results. Probability density plots for the Neoproterozoic-to-Cretaceous portions of the detrital zircon spectra for the samples. Sample GA3081573 had zircons with different provenance to the other two samples. GA3081571 is red, GA3081572 is green, and GA3081573 is blue.

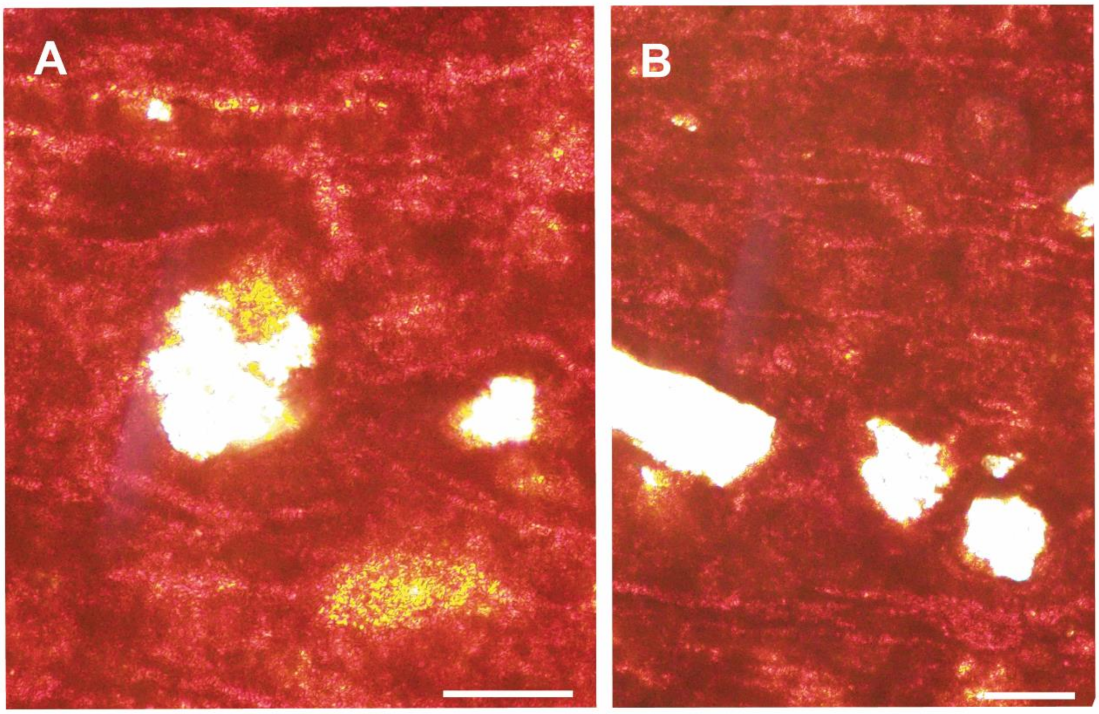

Fig. S5. Two thin-sections through fossiliferous layers from McGraths Flat. The matrix contains little silica, but when grains are present, the goethite forms thinly bedded layers around the grains. Scale bars, 50 µm (A) and 200 µm (B).

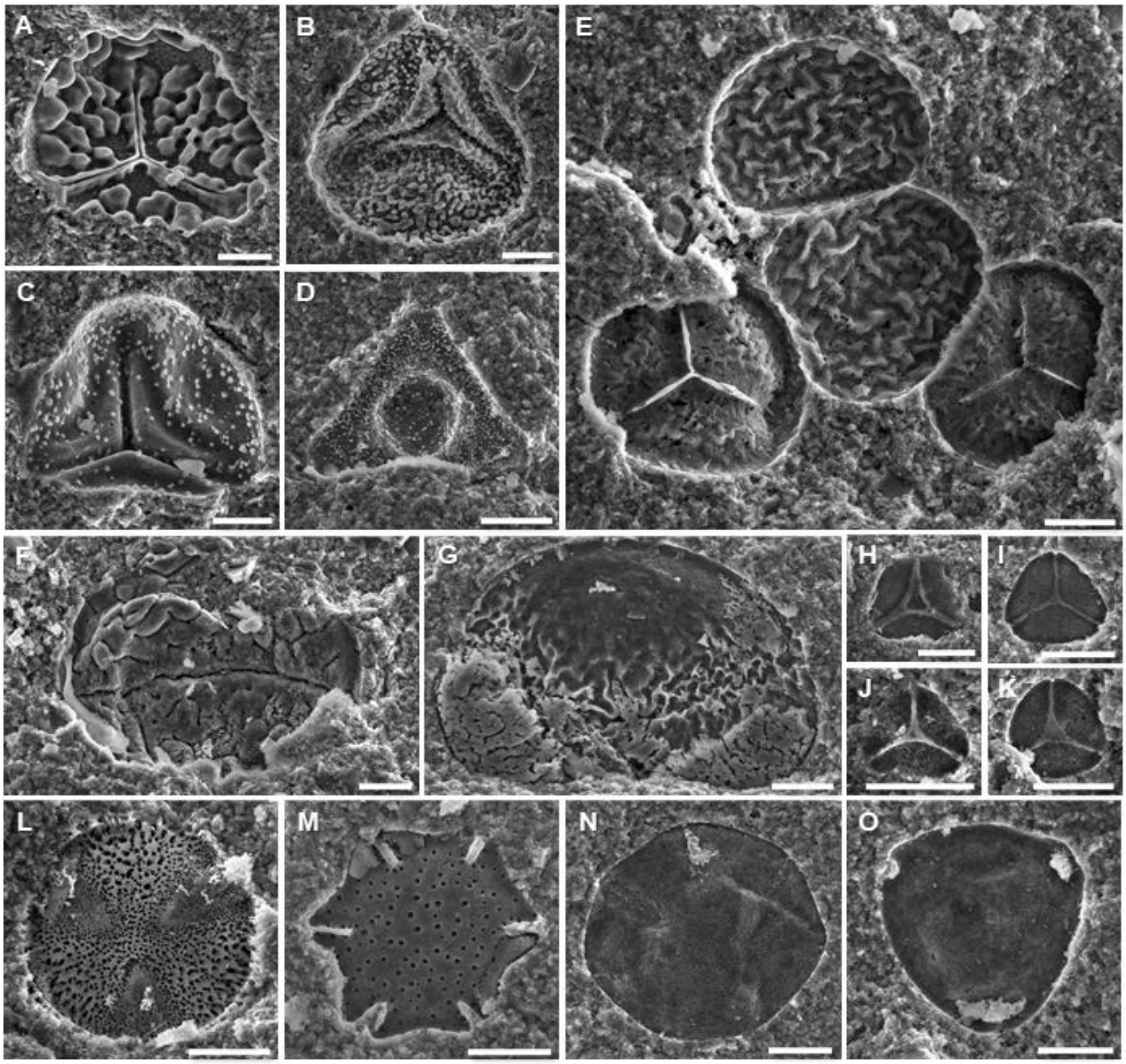

Fig. S6. Biostratigraphically informative spores and pollen. (**A**) *Rugulatisporites trophus* Stover & Partridge (AM F.145717-P33); (**B**) *Rugulatisporites cowrensis* (Martin) Mildenhall & Pocknall (AM F.146023-P30); (**C**) *Foveotrilites balteus* Stover & Partridge (AM F.146164-P19); (**D**) *Proteacidites pachypolus* Cookson & Pike (AM F.146088-P25); (**E**) *Cingulasporites ornatus* Martin (AM F.146156-P42); (**F**) *Polypodiisporites usmensis* (van der Hammen) Khan & Martin (AM F.146159-P26); (**G**) *Lygistepollenites florinii* (Cookson & Pike) Stover & Evans (AM F.146023-P14); (**H** and **I**) I *Myrtaceidites* ssp. (AM F.146023-P20 and AM F.146157-P17, respectively); (**J** and **K**) *Cupanieidites* ssp. (AM F.146160-P27 and AM F.146088-P42, respectively); (**L**) Margocolporites *vanwijhei* Germeraad et al. (AM F.146163-P23); (**M**) *Nothofagidites emarcidus-heterus* (Cookson) Harris (AM F.146023-P27); (**N**) *Nothofagidites asperus* Romero (AM F.146026-P5); and (**O**) *Haloragacidites harrisii* (Couper) Harris (AM F.145848-P5). Scale bars, 10 μm.

Table S1. XRD results

	**AM F.145277 (fossil)***	**AM F.145277 (matrix)***	**AM F.145330 (matrix)**
**XRD scan**			
X2019062	Goethite		
X2019063			Goethite
X2019064		Goethite, very minor quartz	
X2019065		Goethite, very minor quartz	
X2019066		Goethite	
X2019067			Goethite
X2019068	Goethite		

*Two samples (representing matrix and fossil) were taken from the same specimen.

Table S3. Macroflora and palynomorphs

Fungi		
		Dactylaria
		Dicellaesporites sp.
		Dyadosporites sp.
Bryophyta		
Hepaticae		
		Cingulatisporites ornatus Martin 1973
Sphagnaceae		
		Stereisporites spp.
Lycophyta		
Sellaginellaceae		
		Densoisporites sp.
		Foveotriletes balteus Partriage 1973
Filicopsida		
Incertae sedis		
		Verrucosisporites sp. (fern spore)
		Laevigatosporites sp. (numerous monolete-producing ferns including Blechnaceae)
		Cyathidites spp.
		Matonisporites sp.
Cyatheaceae		
	Cyathea	
		Cyathidites palaeospora (Martin) Alley & Broadbridge 1992
Schizeaceae		
	Lygodiaceae	
		Lygodium sp.
		Cyathidites splendens Harris 1965
Lophosoriaceae		

Cyatheacidites annulatus Cookson 1947

Dicksoniaceae/Gleicheniaceae

Dictyophyllidites spp.

Gleicheniaceae

Gleichenia sp.

Gleicheniidites sp.

Osmundaceae

Osmundacidites sp.

Blechnaceae

cf. *Stenochlaena*

Polypodiisporites usmensis (Van der Hammen) Khan & Martin 1971

Thrysopteridaceae

Calochlaena-Culcita

Rugulatisporites cowrensis (Martin) Mildenhall & Pocknall 1989

Rugulatisporites mallatus Stover & Partridge 1973

Rugulatisporites trophus Stover & Partridge 1973

Hymenophyllaceae

Hymenophyllum sp.

Pteridospermaphyta

Alisporites sp. (probably recycled from underlying Mesozoic sediments)

Coniferophyta

Araucariaceae

Araucaria

Araucariacites australis Cookson 1947

Dilwynites sp.

Agathis sp.

Podocarpaceae

Lygistepollenites florinii (Cookson & Pike) Stover & Evans 1973

Podocarpidites ellipticus Cookson 1947

Phyllocladidites mawsonii Cookson. 1947

Podocarpus sp.

Dacrydium sp.

Monocotyledonae

Incertae sedis

Monocot leaves

Sparganiaceae

Aglaoreidia sp.

Poaceae

Graminidites sp.

Restionaceae

Milfordia spp.

Dicotyledonae

Anarcardiaceae

Ailanthipites paenestriatus (Stover & Partridge) Milne 1988

Chenopodiaceae

Chenopodipollis cf. *chenopodiaceoides* (Martin) Truswell 1985

Sapindaceae

Cupanieae

Cupanieidites orthoteichus Cookson & Pike 1954

Cupanieidites spp.

Casuarinaceae

Haloragacidites harrisii (Couper) Harris 1971

Gymnostoma sp.

Nothofagaceae

Nothofagus sp.

Nothofagus sp.

Lophozonia

Nothofagidites asperus Romero 1973

Brassospora

Nothofagidites deminutus (Cookson) Stover & Evans 1973

Nothofagidites emarcidus/heterus (Cookson) Harris 1965

Malvaceae

Brachychiton sp.

Euphorbiaceae

Malvacipollis spp.

Caesalpiniaceae

Margocolporites vanwijhei Germeraad, Hopping & Muller 1968

Myrtaceae

Myrtaceidites spp.

Trimeniaceae

Periporopollenites demarcatus Stover & Partridge 1973

Proteaceae

Propylipollis sp.

Proteacidites pachypolus Cookson & Pike 1954

Proteacidites pseudomoides Stover & Partridge 1973

Proteacidites spp.

Banksia sp.

Paracryphiaceae

Quintinia

Quintiniapollis cf. *psilatispora* (Martin) Mildenhall & Pocknall 1989

Santalaceae

Santalum

Santalumidites sp.

Lauraceae

Laurophyllum sp.

Cinamonum sp.

Table S4. Fauna

The number of specimens of each taxon collected to date are provided in brackets.

Insecta

Odonata

Corduliidae indet. (3)

Gomphidae

Austrogomphus sp. (13)

Ephemeroptera

Baetidae

cf. *Platybaetis* sp. (22)

Ichthybotidae or Coloburiscidae indet. (1)

gen. sp. nov. (23)

Hemiptera

Fulgoridae

gen. sp. nov. (1)

Reduviidae

gen. sp. nov. (3)

Cicadidae

cf. Psaltoda sp. (5)

Blattodea

Mastotermitidae

gen. sp. nov. (2)

Megaloptera

Sialidae

gen. sp. nov. (12)

Coleoptera

Chrysomeloidea indet. (14)

Dytiscidae

gen. sp. nov. (1)

Archostemata

gen. sp. nov. (2)

Curculionidae

gen. sp. nov. (1)

Diptera

Limoniidae

Limnophilinae

cf. *Tonnoirella* gen. sp. nov (1)

indet (1)

Chironomidae

Riethia sp. (>115)

Orthocladiinae indet. (5)

Podonominae indet. (3)

Ceratopogonidae indet. (3)

Chaoboridae

Chaoborus sp. nov. (>180)

Hymenoptera

Tenthredinoidea

gen. sp. nov. (1)

Formicidae indet. (5)

indet. (2)

Trichoptera

Polycentropodidae indet. (33)

Arachnida

Araneae

Theridiidae indet. (6)

Mygalomorphae indet. (1)

indet. (6)

**Bivalvia**
Unionida indet. (1)
**Aves indet.**
Aves indet. (1)
**Actinopterygii**
Osmeriformes
cf. Retropinnidae
gen. sp. nov. (25)
Perciformes indet (1)

Table S5. CLAMP physiognomic scores for the flora from McGrath Flat.

Feature	Score
Lobed	4.081633
No Teeth	58.33333
Regular teeth	41.66667
Close teeth	6.25
Round teeth	35.41667
Acute teeth	6.25
Compound teeth	0
Nanophyll	2.564103
Leptophyll 1	0
Leptophyll 2	0
Microphyll 1	7.692308
Microphyll 2	7.692308
Microphyll 3	33.33333
Mesophyll 1	35.89744
Mesophyll 2	10.25641
Mesophyll 3	2.564103
Emarginate apex	0
Round apex	9.090909
Acute apex	59.09091
Attenuate apex	31.81818
Cordate base	0
Round base	21.42857
Acute base	78.57143
L:W <1:1	0
L:W 1-2:1	18.91892
L:W 2-3:1	27.02703
L:W 3-4:1	37.83784
L:W >4:1	16.21622
Obovate	3.947368
Elliptic	78.94737
Ovate	17.10526
